# Rapid eye and hand responses in an interception task are differentially modulated by context-dependent predictability

**DOI:** 10.1167/jov.24.12.10

**Published:** 2024-11-18

**Authors:** Jolande Fooken, Parsa Balalaie, Kayne Park, J. Randall Flanagan, Stephen H. Scott

**Affiliations:** 1 Centre for Neuroscience Studies, Queen's University, Kingston, ON, Canada; 2 Department of Psychology, Queen's University, Kingston, ON, Canada; 3 Department of Biomedical and Molecular Sciences, Queen's University, Kingston, ON, Canada; 4 Department of Medicine, Queen's University, Kingston, ON, Canada; 5 Department of Psychology and Centre for Cognitive Science, Technical University of Darmstadt, Darmstadt, Germany

**Keywords:** interception, eye–hand coordination, reaction time, vigor

## Abstract

When catching a falling ball or avoiding a collision with traffic, humans can quickly generate eye and limb responses to unpredictable changes in their environment. Mechanisms of limb and oculomotor control when responding to sudden changes in the environment have mostly been investigated independently. Here, we investigated eye–hand coordination in a rapid interception task where human participants used a virtual paddle to intercept a moving target. The target moved vertically down a computer screen and could suddenly jump to the left or right. In high-certainty blocks, the target always jumped; in low-certainty blocks, the target only jumped in a portion of the trials. Further, we manipulated response urgency by varying the time of target jumps, with early jumps requiring less urgent responses and late jumps requiring more urgent responses. Our results highlight differential effects of certainty and urgency on eye–hand coordination. Participants initiated both eye and hand responses earlier for high-certainty compared with low-certainty blocks. Hand reaction times decreased and response vigor increased with increasing urgency levels. However, eye reaction times were lowest for medium-urgency levels and eye vigor was unaffected by urgency. Across all trials, we found a weak positive correlation between eye and hand responses. Taken together, these results suggest that the limb and oculomotor systems use similar early sensorimotor processing; however, rapid responses are modulated differentially to attain system-specific sensorimotor goals.

## Introduction

The ability to interact with moving objects in dynamic and unpredictable environments requires complex coordination between the eyes and hands. In a game of table tennis, players track the rapidly moving ball with their eyes and predict the trajectory of the ball after it hits the table to successfully intercept it. In such dynamic tasks, eye and hand movement control is shaped by how certain the available sensory information is and how urgently the motor response has to be executed. For example, there can be uncertainty in the direction the ball will travel following the initial bounce on the table (i.e., if the opponent puts a spin on the ball). There can also be variability in urgency, as the opponent can hit the ball farther away from or nearer to the player, thus requiring less or more urgent responses, respectively. Thus, goal-directed responses often require rapid updating of visual and proprioceptive feedback to ensure successful motor actions.

In everyday action tasks, eye and hand movements have been shown to be coordinated in stereotypical ways (Land, 2006). In stationary reaching, eye movements typically lead hand movements, fixating on key landmarks and objects before they are manipulated (de Brouwer, Flanagan, & Spering, 2021; Johansson, Westling, Bäckström, & Flanagan, 2001). This relationship is more complex when interacting with moving targets. Intercepting a moving target involves predicting where the target will be at future states (Bosco et al., 2015; Fooken, Kreyenmeier, & Spering, 2021; Zago, Iosa, Maffei, & Lacquaniti, 2010). This prediction is crucial to overcome the inherent delays from visual and proprioceptive afferents required to generate goal-directed motor actions in the sensorimotor system (Brenner & Smeets, 2018; Cross, Cook, & Scott, 2024; Ghez, Gordon, & Ghilardi, 1995; Todorov & Jordan, 2002; Zago, McIntyre, Senot, & Lacquaniti, 2009). During interception tasks, maintaining gaze on the moving target (via smooth pursuit eye movements) allows continuous updating of the trajectory of the target that can aid motion prediction (Spering, Schütz, Braun, & Gegenfurtner, 2011) and manual interception performance (Fooken, Yeo, Pai, & Spering, 2016; Goettker, Brenner, Gegenfurtner, & de la Malla, 2019; Kreyenmeier, Kämmer, Fooken, & Spering, 2022; Mrotek, 2013; Mrotek & Soechting, 2007). However, eye movement behavior is also affected by the predictability of the path of the moving object and visual certainty. In situations of high uncertainty, such as when the moving target bounces or is occluded, observers shift their gaze away from the moving object to the anticipated interception locations (de la Malla, Rushton, Clark, Smeets, & Brenner, 2019; Diaz, Cooper, Rothkopf, & Hayhoe, 2013; Fooken & Spering, 2019; Mann, Nakamoto, Logt, Sikkink, & Brenner, 2019). Thus, the coordination between eye and hand movements can be modulated by visuomotor task demands.

Whereas a close coordination between eye and hand movements has been observed in action tasks, such as object manipulation or manual interception, the link between eye and hand movement control in tasks requiring rapid visuomotor responses is less clear. Previous research has shown that humans are able to elicit very rapid motor responses to sudden visual stimuli—a phenomenon referred to as express visuomotor responses or stimulus locked responses (Corneil, Munoz, Chapman, Admans, & Cushing, 2008). Express visuomotor responses have been found in the generation of saccadic eye movements (Dorris, Paré, & Munoz, 1997), as well as in the upper limb responses following visual target appearance (Pruszynski et al., 2010). For upper limb responses, it has been shown that high certainty about a forthcoming perturbation and temporal predictability of perturbation onset evokes reliable and rapid muscle responses (Contemori, Loeb, Corneil, Wallis, & Carroll, 2021; Kozak, Cecala, & Corneil, 2020). Early waves of upper limb muscle responses are also modulated by the level of response urgency, with movement corrections occurring earlier relative to the moment of perturbation when the time available to make a response is limited (Crévécoeur, Kurtzer, Bourke, & Scott, 2013; Maurus, Jackson, Cashaback, & Cluff, 2023; Poscente, Peters, Cashaback, & Cluff, 2021). The fact that express visuomotor responses are sensitive to task context supports the idea that subcortical control mechanisms of rapid movement responses are modulated by top–down cortical inputs (Contemori, Loeb, Corneil, Wallis, & Carroll, 2023).

Here, we examined eye and hand responses to visual perturbations during a rapid interception task. Participants used a virtual paddle to intercept a target moving down a vertically oriented computer screen. In the majority of trials, the moving target was visually perturbed (jumped), suddenly shifting spatial location to the left or right and continuing moving down after the jump. To examine the effect of jump certainty, we modified the frequency at which the target jumped. In the high-certainty condition, the target jumped to the left or right of the midline in 100% of trials. In the low-certainty condition, the target jumped in 60% of the trials and continued to move straight down the midline in 40% of trials (no-jump trials). We further examined the effect of response urgency, by varying the onset of jumps across all trials. Early jumps required a low level of urgency (responses within 450 ms), middle jumps required a medium level of urgency (responses within 350 ms), and late jumps required a high level of urgency (responses within 250 ms).

**Figure 1.**
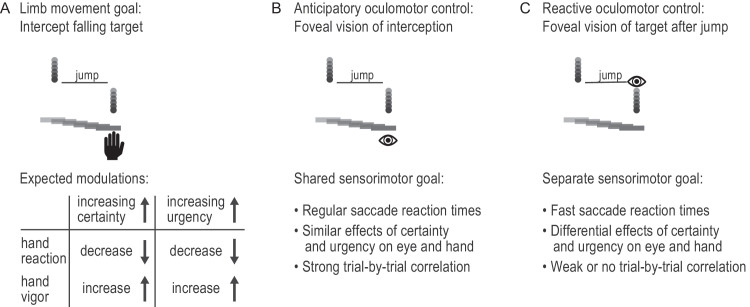
Potential effects of jump certainty and response urgency on eye and hand responses. (**A**) Hand movements generated to intercept a falling target are expected to be earlier and faster in high-certainty and more urgent trials. (**B**) The oculomotor system may share the sensorimotor goal of the upper limb motor system (anticipatory control for interception), in which case the effects of jump certainty and response urgency will be similar. (**C**) Alternatively, the goal of the oculomotor system may be to detect the target jump (reactive control), in which case eye movements will be differentially affected by changes in jump certainty and response urgency.

Using this paradigm, we had two expectations of how jump certainty and response urgency would affect hand responses (Figure 1A). First, we hypothesized that high-certainty blocks would be associated with earlier (lower reaction time) and faster (higher vigor) hand responses compared with low-certainty blocks. Second, we hypothesized that, as the response urgency increased, hand responses would be initiated earlier (lower reaction time) and with greater vigor. However, the goal of the oculomotor system is less clear (Figure 1B). An anticipatory saccade that moves the eye to the location of interception before the target and the hand arrive could provide foveal vision to guide contact between the paddle and the target (Fooken et al., 2021). In this case, we would predict regular saccade latencies of ∼200 ms (Carpenter & Williams, 1995), and similar modulations of eye reaction times and vigor as observed in hand responses for varying levels of certainty and urgency. Moreover, eye and hand responses would be strongly correlated on a trial-by-trial basis (Figure 1B). Alternatively, eye responses in this task could be reactive; that is, the eyes are rapidly shifted to the new target position following the target jump. In this case, we would predict eye responses with latencies similar to express saccades (Fischer & Ramsperger, 1984; Paré & Munoz, 1996). Moreover, we would expect a differential modulation of eye and hand responses by jump certainty and response urgency and only a weak or no correlation between eye and hand responses (Figure 1C).

## Methods

### Participants

A total of 14 healthy individuals (12 right-handed; 11 females; mean age, 26.5 years; age range, 18–44 years) participated in the experiment. This sample size was chosen based on pilot work finding consistent effects of jump certainty and response urgency on limb kinematics in 16 individuals. All participants had no self-reported neurological or musculoskeletal impairments and had normal or corrected-to-normal vision. The study was approved by the Queen's University Health Sciences & Affiliated Teaching Hospitals Research Ethics Board (TRAQ no. 6003707) and adhered with the tenets of the Declaration of Helsinki. Participants gave written informed consent and were compensated with a small honorarium ($10 CAD).

### Apparatus

**Figure 2.**
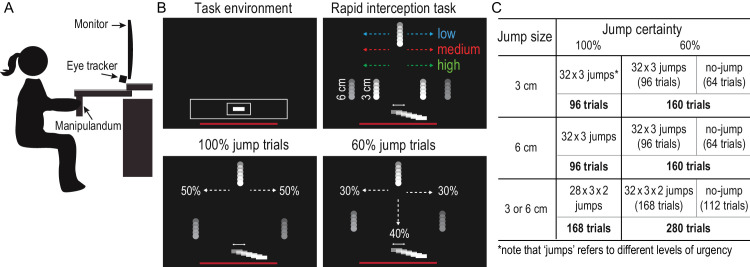
Experimental setup and conditions. (**A**) Participants moved the handle of a robotic manipulandum in the horizontal plane to intercept moving targets viewed on a vertical screen. (**B**) Each trial started by showing the task environment (top left). The response urgency was manipulated by changing the position at which the target jumped (top right). The target could jump 3 cm, 6 cm, or both within a block. In the high-certainty condition (100% jump trials; lower left), the target moved down the midline before it randomly jumped to the left or right side (lower left). In the low-certainty condition, the target could continue to move down the midline (no-jump) in 40% of the trials (lower right). (**C**) Participants performed 32 or 28 interceptions for each urgency level in different blocks of jump certainty and jump size combinations.

Experiments were conducted using the Kinarm End-Point robot (Kinarm, Kingston, ON, Canada). Participants were seated in chairs that supported their backs and used one hand to grasp the handle of the robotic manipulandum, which allowed movement along the horizontal plane. Movements made by the participant were tracked by the manipulandum and presented as a cursor on a 32-inch vertical display placed 37 cm from the participant (Figure 2A). Note that, at the chosen viewing distance, 1 cm on the screen corresponded to 1.5 visual degrees (deg). The mapping between the handle and cursor movement was the same as a standard computer mouse, such that forward and backward movements of the handle moved the cursor up and down, and left and right handle movements moved the cursor left and right. Movements of the handle were recorded at a sampling rate of 1000 Hz. The inherent visual display delay was accounted for by using the latency reported by the graphics card in the robot computer and the calculated refreshing latency (∼50 ms). The position of a participant’s right eye was recorded using a video-based eye tracker (EyeLink; SR Research, Kanata, ON, Canada) with a sampling rate of 500 Hz. A combined chin and forehead rest minimized head movements during the experiment.

### Experimental protocol

We modified a previously established interception task to assess the coordination between eye and hand responses (Park, Ritsma, Dukelow, & Scott, 2023). Figure 2B illustrates the rapid interception task, in which participants intercepted a target moving down a vertical screen with a virtual paddle (white rectangle, 2 × 0.5 cm) representing the position of the hand. At the beginning of each trial, participants were shown the task environment, which consisted of the workspace represented by a white box (workspace, 28 × 3.5 cm) indicating the allowed area of movement. A smaller box (starting area, 3.5 × 2 cm) indicated the area into which participants had to move their paddle to begin a trial. A red line representing the bottom limit for intercepting the moving target was displayed to deter individuals from moving below the workspace. After holding the paddle in the starting area for 200 ms, the task environment disappeared, and a target (white circle with a radius of 0.5 cm) appeared at the top of the screen (22 cm from the center of the starting area). The target then immediately started to move down the screen at a speed of 25 cm/s. The target either continued to move straight down or could be visually perturbed to the left or right with the same probability. After the perturbation, the target continued to move straight down. Participants had to intercept the target with any part of the paddle and in any direction before it reached the bottom of the workspace. If a participant left the workspace that was shown at the beginning of the trial (the large white box), the robot applied a step force in the opposite direction of their hand movement in order to push their hand back into the box. On successful interceptions, haptic feedback was applied to the participant's hand via the robotic manipulandum to indicate success. Subsequent trials began 500 ms after the target was intercepted or missed. Participants were free to move their eyes naturally (i.e., no instructions about eye movements were given).

To investigate the relationship between eye and hand movements, we manipulated jump certainty and response urgency. We examined certainty by having two groups of blocks of either high-certainty conditions (target jumped in 100% of the trials) or low-certainty conditions (target jumped in 60%). We probed response urgency by varying the onset of visual perturbation. The target was perturbed at three different locations from the top of the workspace, requiring more urgent responses for later target jumps. The highest jump position was 11.25 cm above the starting area (low urgency; ∼450 ms to respond), the middle jump position was 8.75 cm above the starting area (medium urgency; ∼350 ms to respond), and the lowest jump position was 6.25 cm away (high urgency; ∼250 ms to respond). Response urgency (i.e., jump onset position) was randomized at equal probability across all trials. Further, we examined the magnitude of motor responses by varying the size of the jump (3 cm, 6 cm, or both 3 cm and 6 cm with equal probability). The size of the jump was varied in three separate blocks, and for each jump size we tested both high and low certainty, resulting in six total blocks (Figure 2C). Within each certainty condition, the order of blocks with different jump sizes was randomized. Participants completed either three high-certainty blocks and then three low-certainty blocks or three low-certainty blocks and three high-certainty blocks. Before each block, the eye tracker was calibrated using the standard EyeLink five-point calibration procedure. There were 960 trials in total, which took participants about 1 hour to complete, including breaks.

### Eye and hand movement analysis

Eye and hand movement data were analyzed offline using custom codes in MATLAB R2021a (MathWorks, Natick MA). The *x* and *y* positions of the center of the robotic handle were used for hand movement analysis. The position of the handle was filtered using a third-order, zero-phase lag, 20-Hz Butterworth filter. We analyzed horizontal and vertical interception position, defined as the *x* and *y* positions of the robotic handle at the time the target first contacted any part of the paddle. We further analyzed hand movement reaction time and vigor. Hand movement reaction time was determined using the extrapolation method (Oostwoud Wijdenes, Brenner, & Smeets, 2014; Veerman, Brenner, & Smeets, 2008). In brief, we calculated the time at which a line that was fitted through 25% and 75% of the hand peak velocity intersected with the zero-velocity point. Hand movement vigor was calculated similar to movement vigor previously reported in the literature (Reppert, Lempert, Glimcher, & Shadmehr, 2015). In each trial, we defined the hand movement amplitude as the absolute farthest distance the hand traveled between the time of target movement onset and the time of target interception, or the time the target left the interception zone in target-miss trials. We then fitted a hyperbolic function for each participant (*n*) to capture the relationship between movement peak velocity ν*_n_* and amplitude *x*:

(1) $$\begin{array}{c} v_{n}=\alpha_{n}\left(1-\frac{1}{1+\beta_{n}x}\right)\; \end{array}$$

This fit yielded participant-specific parameters of α*_n_* and β*_n_* that were used to calculate the expected movement velocity (ν*_fit_*) given each observed movement amplitude. Vigor was defined as a within-participant measure by comparing the actual movement velocity with the expected movement velocity (ν*_n_*/ν*_fit_*). Thus, a vigor value >1 indicates that a given movement was faster than expected, and a value <1 indicates that the movement was slower than the average movement for this amplitude.

The *x* and *y* eye positions of the right eye were sampled in screen-centered coordinates, and position signals were filtered using a second-order 15-Hz Butterworth lowpass filter. We analyzed vertical and horizontal eye positions at selected times throughout the task (time of target movement onset, at the time of target jump, and 250 ms after target jump). We further analyzed the eye movement accuracy, reaction time, and vigor of the first saccade that occurred at least 50 ms following the target jump. Akin to hand movement measures, reaction time was defined as the difference from the time the visual target jumped to the time of saccade onset. Eye movements were labeled as saccades when five consecutive samples of the filtered eye velocity exceeded a fixed velocity criterion of 30 cm/s. Saccade onsets and offsets were labeled as the time that the sign of the acceleration signal reversed either before eye velocity exceeded (saccade onset) or was less than (saccade offset) the velocity threshold (Fooken & Spering, 2019). To determine eye movement vigor, we followed the same procedure as outlined for hand movement vigor. Saccade accuracy was defined as the *x* and *y* distances between the eye and the target position at the time of saccade offset.

### Statistical analysis

To evaluate the effects of jump certainty and response urgency on hand and eye movement measures, we calculated the median value for each condition and participant. We then used repeated-measures analyses of variance (ANOVAs) with an α level of 0.05. Post hoc comparisons were done using two-sided, paired *t*-tests with Bonferroni correction. Because we did not have an a priori hypothesis of how jump size (3 cm or 6 cm) would affect selected eye and hand measures, we averaged across jump size unless stated otherwise in the manuscript. Statistical tests were conducted using R (R Foundation for Statistical Computing, Vienna, Austria).

To investigate the trial-by-trial correlation between eye and hand measures, we conducted a linear mixed model (LMM) analysis using the *lme4* package for R (Bates, Mächler, Bolker, & Walker, 2015). Specifically, we treated the hand measure (i.e., reaction time or vigor) as the continuous outcome variable, the eye measure as a continuous predictor, and experimental manipulations (i.e., certainty and urgency) as categorical predictors. We considered jump certainty and response urgency to be fixed effects and the eye measure a fixed and random effect per participant:

(2) $$\begin{array}{ccc} & & measure_{hand}\;∼\;1+\;measure_{eye}*certainty\\ & & \;*\;urgency+(1+measure_{eye}\;|\;participant)\; \end{array}$$

Predictor values were standardized using the R function *scale*.

### Data exclusion

One participant was excluded due to a high loss of eye data, affecting 47% of their trials. Across the remaining participants’ trials, 2034 out of 13,440 (15%) were flagged. Trials were flagged if the eye signal was lost (e.g., due to blinks) in the time window from target movement onset to the time of interception (6.5%) or if the participant would move preemptively (8.5%). We labeled hand movements as preemptive if the participant's reaction time was lower than 120 ms after the target jumped or if they moved to the opposite side of the jump direction.

## Results

The goal of this experiment was to investigate the coordination of eye and hand responses when intercepting a rapidly moving target that could jump at a variable time to the left or right. In a given block, the target jumped with either high certainty (100% jump trials) or low certainty (60% jump trials). In no-jump trials (40% of all trials in the low-certainty condition), the target continued to travel down the midline, and participants were required to keep the paddle at the center of the screen to intercept the target successfully. We manipulated response urgency by changing the time at which the target jumped (see Figure 2B).

### Patterns of eye and hand movements were similar in jump and no-jump trials

**Figure 3.**
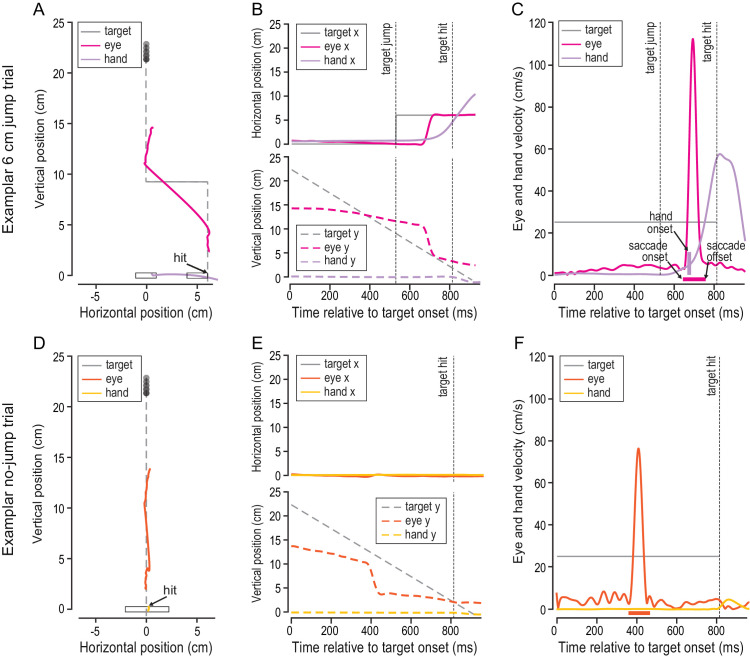
Eye and hand movement responses in the rapid interception task. (**A**) Eye (dark red), hand (light red), and target (black) position in screen-centered coordinates in a representative jump trial. (**B**) Horizontal eye, hand, and target position (top panel) and vertical eye, hand, and target position (bottom panel) across time. The target jump and target interception times are indicated by dashed vertical lines. (**C**) Eye, hand, and target velocity across time. Saccade onset and offset of the targeting saccade following the target jump are indicated by arrows and the thick horizontal bar. Hand movement onset is indicated by the thick vertical bar. (**D**) Eye (dark purple), hand (light purple), and target (black) position in screen-centered coordinates in a representative no-jump trial. (**E**) Horizontal eye, hand, and target position (top panel) and vertical eye, hand, and target position (bottom panel) across time. The time of the target interception is indicated by the dashed vertical line. (**F**) Eye, hand, and target velocity across time.

The rapid interception task elicited a combination of tracking and saccadic eye movements. Figure 3A shows the two-dimensional (2D) eye, hand, and target position of a representative high-certainty trial (Figure 3A). Note that all positional data are referenced to the start position of the paddle; that is, the origin of the coordinate system (0, 0) indicates the center of the starting area (Figure 2B). Eye, hand, and target positions (Figure 3B) and velocities (Figure 3C) for the same trial are also shown across time. In this trial, the participant looked at the center of the screen and waited for the target to move down the midline. The participant briefly tracked the target with smooth pursuit eye movements before initiating a reactive saccade toward the target after it jumped to the right.

The lower panels in Figure 3 show the eye, hand, and target positions of a representative low-certainty trial—in which the target did not jump—in a screen-centered reference frame (Figure 3D) and across time (Figure 3E). Eye, hand, and target velocities of the same trial are also shown across time (Figure 3F). Similar to the perturbation trial, the participant looked at the center of the screen at the beginning of the trial. The participant tracked the target with smooth pursuit and a catch-up saccade until it was intercepted by keeping the paddle on the midline.

Across all trials and participants and in both certainty conditions (including jump and no-jump trials), we found a consistent pattern of eye movements. In the time window from target movement onset to the time of target jump or target interception for no-jump trials, participants made no saccades in 42% of the trials, made a single saccade in 47% of the trials, and elicited more than one saccade in 11% of the trials. Eye velocity in the same time window was on average 7.58 ± 0.97 cm/s (mean eye velocity and standard error across participants), which was much lower than the target velocity of 25 cm/s, indicating that participants did not smoothly pursue the moving target prior to a target jump. Following a target jump, participants initiated a reactive saccade in 93% of all trials. Of note, in a majority of these trials (96%) participants did not make another saccade before they intercepted the moving target. The described eye movement patterns show that, whereas eye movements prior to target jump were quite variable (short periods of pursuit, catch-up saccades, or fixation), a single reactive saccade was elicited following a target jump in almost every trial.

### Eye movement position was modulated by the response urgency

**Figure 4.**
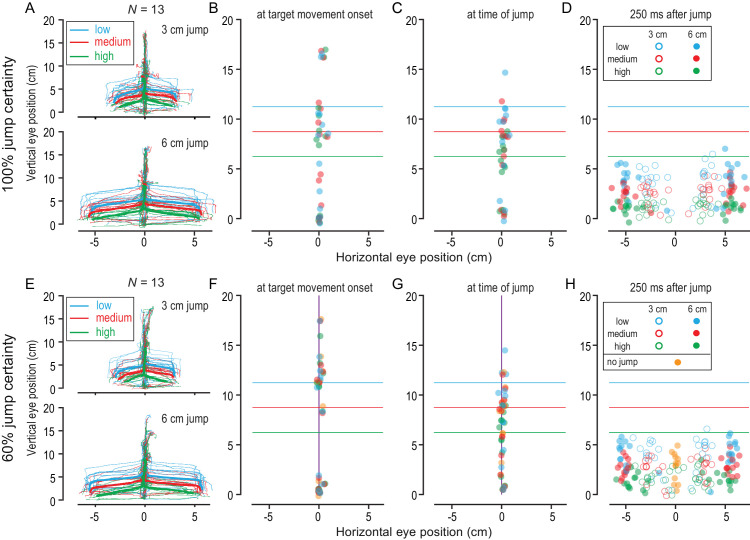
Eye responses in a rapid interception task. (**A**) Gaze position in 100% certainty trials averaged across low (blue), medium (red), and high (green) urgency trials of each participant (thin lines) and averaged across the group (thick lines) separately plotted for the 3-cm (top) and 6-cm (bottom) target jump size conditions. (**B**–**D**) The average 2D eye position is shown for each participant and each urgency level at the time of the target movement onset (**B**), the time of the target jump (**C**), and 250 ms after the target jump (**D**). (**E**–**H**) Corresponding plots for the 60% jump certainty condition. Eye positions in no-jump trials are indicated in purple.

Figure 4 illustrates the overall gaze pattern observed in our experiment. Figure 4A shows the gaze position (from target movement onset to target interception) of all participants (thin lines) in screen-centered coordinates averaged across high-certainty 3-cm (top) and 6-cm (bottom) jump trials. The gaze position averaged across participants at different urgency levels is indicated by thick blue (low urgency), red (medium urgency), and green (high urgency) lines. Figure 4E shows the corresponding screen-centered gaze position in low-certainty trials. To further describe the observed gaze pattern, we compared participants’ eye positions at three distinct time points: (1) at the time of target movement onset (Figures 4B and 4F), (2) at the time of target jump (Figures 4C and 4G), and (3) 250 ms after the time of jump (Figures 4D and 4H). We chose 250 ms to allow sufficient time for saccades to land following the target jump.

We first compared the effect of certainty (high vs. low) and urgency (low, medium, or high) on vertical eye position at the three selected time points using a repeated-measures 2 × 3 ANOVA. We found no effect of certainty or urgency and no interaction for vertical eye position at the time of target movement onset (*F* < 1.7, *p* > 0.2, η < 0.13). We also found no effect of certainty and no interaction on vertical eye position at the time of the target jump (*F* < 0.7, *p* > 0.5, η < 0.06). We found an effect of urgency on vertical eye position at the time of target jump, *F*(2, 24) = 20.72, *p* < 0.001, η = 0.63. Finally, we did not find an effect of jump certainty (*F* < 1.5, *p* > 0.2, η < 0.12) on vertical eye position 250 ms after target jump, but did find a significant effect of urgency, *F*(2, 24) = 91.82, *p* < 0.001, η = 0.88, and a significant interaction between jump certainty and response urgency, *F*(2, 24) = 6.52, *p* = 0.005, η = 0.35. Taken together, these results indicate that vertical eye position was significantly affected by target jump times, with vertical eye position being lower as the urgency level increased. Vertical eye position was not affected by jump certainty. As illustrated by each participant's gaze position (Figures 4A and 4E), the between-participants variability was high at target movement onset (∼6 cm), but participants converged to similar vertical gaze positions following the target jump.

Horizontal gaze positions were near the midline until after the target jumped (Figures 4B, 4C, 4F, and 4G), and horizontal gaze remained at the midline during trials where the target did not jump (purple dots in Figures 4F to 4H). Following the target jump, the horizontal eye position scaled with the size of the target jump, landing on average 2.65 ± 0.16 cm away from the midline in 3-cm jump trials and 5.29 ± 0.09 cm away from the midline in 6-cm jump trials.

Finally, we investigated the accuracy of the first saccade that occurred after target jump. Whereas horizontal saccade accuracy was unaffected by jump certainty and response urgency, vertical saccade accuracy was affected by jump certainty, *F*(1, 12) = 4.85, *p* = 0.048, η = 0.29, and response urgency, *F*(2, 24) = 123.92, *p* < 0.001, η = 0.91. Post hoc comparisons showed that participants were on average very accurate in high-certainty blocks (vertical saccade error, –0.08 ± 0.32 cm), but tended to land above, or behind, the actual target position in low-certainty blocks (vertical saccade error, 0.27 ± 0.32 cm). Moreover, saccades were most accurate in medium-urgency trials (vertical saccade error, 0.05 ± 0.19 cm). In low-urgency trials, saccades tended to land below, or ahead, of the moving target position (vertical saccade error, –0.94 ± 0.26 cm), and, in high-urgency trials, saccades tended to land above, or behind, the moving target position (vertical saccade error, 1.19 ± 0.16 cm).

### Interception accuracy decreased with uncertainty and increasing urgency

**Figure 5.**
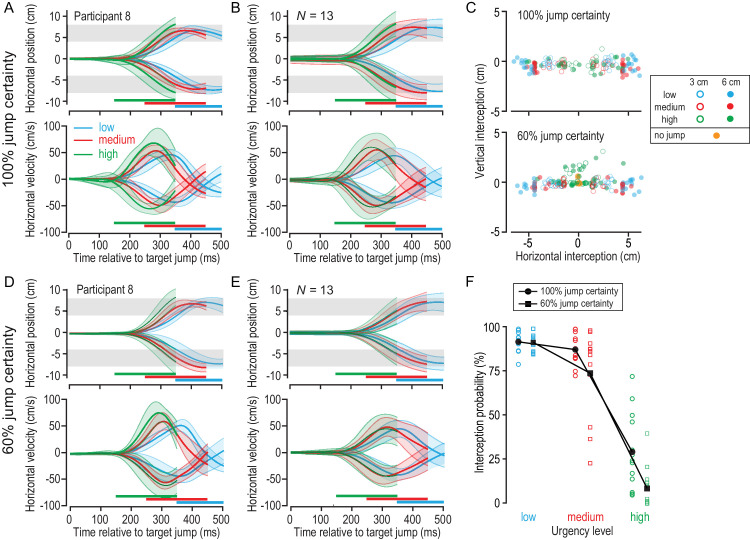
Hand responses in the rapid interception task. (**A**) Horizontal hand position (top) and velocity (bottom) across time for low (blue), medium (red), and high (green) urgency trials shown for an exemplar participant in the high-certainty condition. The gray-shaded areas represent the region in which the paddle could intercept the target. The horizontal, colored bars indicate the time period in which the target had to be intercepted for each urgency level. (**B**) Corresponding plots averaged across all participants in the high-certainty condition. (**C**) Hand position at the time of interception or when the missed target left the screen in the high-certainty (top) and low-certainty (bottom) conditions. Open circles indicate 3-cm jump trials and filled circles indicate 6-cm jump trials. Color denotes urgency level, and purple indicates trials in which the target did not jump. (**D**, **E**) Plots of horizontal hand position and velocity across time for an exemplary participant (**D**) and averaged across all participants (**E**) in the low-certainty condition. (**F**) Probability of intercepting the moving target following a target jump in the high-certainty (circles) and low-certainty (squares) jump conditions and across urgency levels.

Figure 5 illustrates the overall hand movements observed in our experiment. Figures 5A and 5B show the horizontal hand position (upper panels) and absolute hand velocity (lower panels) during high-certainty 6-cm jump trials for an exemplar participant and the average of all participants, respectively. The corresponding plots of the horizontal hand position and velocity during 6-cm low-certainty trials are shown in Figures 5D and 5E. In these plots, hand movements are averaged across different levels of urgency as indicated by color. Because the moving target could be intercepted with any part of the paddle, there is a 4-cm-wide region and a limited time window to intercept the falling target successfully (see gray-shaded regions and horizontal, colored lines in Figures 5A, 5B, 5D, and 5E). We found that the onset of participants’ hand responses scaled with response urgency.

To compare performance across conditions, we analyzed hand position at the time of the target interception and the probability of successfully intercepting the falling target across conditions. Figure 5C shows for each certainty condition the urgency level and jump size, as well as the average screen-centered hand position at the time of the target interception or when the missed target left the screen. Overall, hand movement scaled with the size of the jump, and participants intercepted the target on average 2.02 ± 0.10 cm away from the midline in 3-cm jump trials and 3.95 ± 0.15 cm away from the midline in 6-cm jump trials. The fact that participants undershot the actual target position indicates that they tended to contact the falling target with the outer side of the paddle.

We used a repeated-measures 2 × 3 ANOVA to quantify the effect of certainty and urgency on horizontal interception position. We found an effect of jump certainty, *F*(1, 12) = 30.55, *p* < 0.001, η = 0.72; an effect of response urgency, *F*(2, 24) = 193.09, *p* < 0.001, η = 0.94; and a significant interaction, *F*(2, 24) = 4.18, *p* = 0.028, η = 0.26. The strong effect of response urgency on horizontal interception position reflects the shorter time available for intercepting the perturbed target (see Figures 5A, 5B, 5D, and 5E). Participants intercepted the target with the center of the paddle in low-urgency trials but intercepted the target with the outer edge of the paddle in medium- and high-urgency trials. On average, participants tended to move the paddle slightly downward to intercept the falling object (vertical interception position, –0.27 ± 0.06 cm). Interestingly, we observed that, in the low-certainty and high-urgency conditions, participants tended to move the paddle upward (vertical interception position, 0.34 ± 0.22 cm), as if they expected the target to stay in the middle (see green dots above the center in the lower panel of Figure 5C).

Figure 5F shows the averaged probability of intercepting the falling target across task conditions. Individual color-coded symbols represent the median response value of each participant, and the larger black symbols (connected by black lines) represent the group means. We found an effect of jump certainty, *F*(1, 12) = 24.27, *p* < 0.001, η = 0.67; an effect of response urgency, *F*(2, 24) = 194.12, *p* < 0.001, η = 0.94; and a significant interaction effect, *F*(2, 24) = 5.73, *p* = 0.009, η = 0.32. Although participants successfully intercepted a majority of targets in low-urgency trials (high-certainty, 91.7% ± 1.5%; low-certainty, 91.4% ± 1.4%) and medium-urgency trials (high-certainty, 87.3% ± 2.4%; low-certainty, 73.9% ± 6.7%), they only intercepted about a third of the targets in high-certainty, high-urgency trials (29.2% ± 6.0%) and only a few targets in low-certainty, high-urgency trials (8.5% ± 3.2%).

### Differential effects of certainty and urgency on eye and hand responses

**Figure 6.**
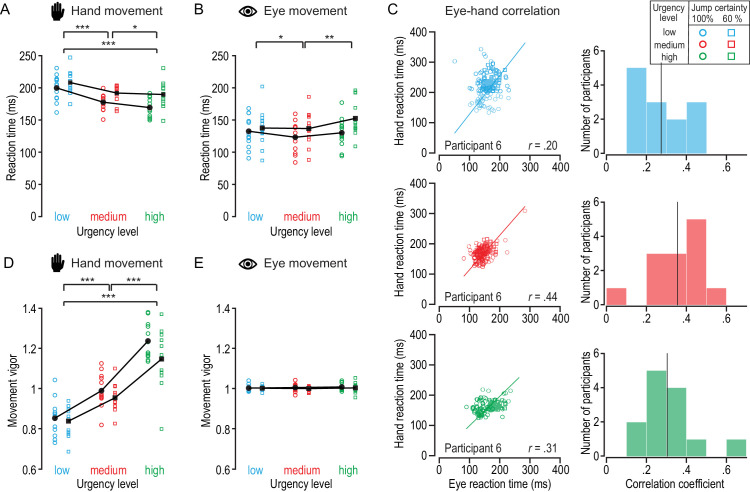
Eye and hand responses across task conditions. (**A**) Hand movement reaction times for different task conditions. Single symbols indicate median reaction times for each participant for high-certainty jumps (circles) and low-certainty jumps (squares), and separate for low (blue), medium (red), and high (green) urgency trials. (**B**) Corresponding eye reaction times. (**C**) Trial-by-trial correlation between eye and hand reaction time for low (top panels), medium (middle panels), and high (bottom panels) urgency trials for a representative single participant (left), and histograms of the correlation coefficients of all participants (right). (**D**, **E**) Average hand vigor (**D**) and average eye vigor (**E**) for different task conditions.

Figure 6 shows the eye and hand reaction times, vigor, and trial-by-trial correlations between eye and hand responses for the two certainty conditions and three urgency levels. To directly compare eye and hand responses, only trials in which a saccade was made (93% of all trials) were included in this analysis. To test the effect of jump certainty and response urgency on eye and hand responses, we used four separate repeated-measures 2 × 3 ANOVAs. Hand responses were, on average, initiated 188.8 ± 11.9 ms after the target jump. We found an effect of jump certainty, *F*(1, 12) = 27.14, *p* < 0.001, η = 0.69, and response urgency, *F*(2, 24) = 17.31, *p* < 0.001, η = 0.59, on hand reaction time, as well as a significant interaction, *F*(2, 24) = 3.87, *p* = 0.04, η = 0.24 (Figure 6A). A post hoc comparison of the two certainty conditions confirmed that participants on average initiated their hand responses 14 ms earlier in the high-certainty blocks compared with the low-certainty blocks, *t*(38) = 6.4; *p_adjust_* < 0.001, *d* = 1.3. Compared to low-urgency trials, hand responses were initiated 19 ms earlier in medium-urgency trials, *t*(25) = 6.9, *p_adjust_* < 0.001, *d* = 1.3, and 25 ms earlier in high-urgency trials, *t*(25) = 5.2, *p_adjust_* < 0.001, *d* = 1.0. Hand movements were on average initiated 5 ms earlier in high-urgency compared with medium-urgency trials, *t*(25) = 2.2, *p_adjust_* = 0.01, *d* = 0.4.

Eye movements preceded hand movements by ∼50 ms and were on average initiated 135.4 ± 6.3 ms after the target jump. We found an effect of jump certainty, *F*(1, 12) = 11.72, *p* = 0.005, η = 0.49, and response urgency, *F*(2, 24) = 5.27, *p* = 0.01, η = 0.31, on eye reaction time, as well as a significant interaction, *F*(2, 24) = 13.48, *p* < 0.001, η = 0.53 (Figure 6B). Post hoc comparisons of the two certainty conditions confirmed that participants on average initiated their eye movements 14 ms earlier in the high-certainty blocks compared with the low-certainty blocks, *t*(38) = 5.0, *p_adjust_* < 0.001, *d* = 0.8. Compared with the medium-urgency trials, eye responses were initiated 5 ms later in low-urgency trials, *t*(25) = 2.8, *p_adjust_* = 0.03, *d* = 0.6, and 11 ms later in high-urgency trials, *t*(25) = 4.1, *p* = 0.001, *d* = 0.8. Thus, whereas hand reaction times were systematically faster for high-urgency levels, eye reaction times were differentially affected.

We investigated the trial-by-trial relationship between eye and hand reaction times using a LMM with eye reaction time as a continuous predictor and certainty and urgency as categorical predictors. Eye reaction time (β *=* 12.12; 95% confidence interval [CI], 9.22–15.13; *p* < 0.001), jump certainty (β *=* 5.71; 95% CI, 4.92–6.49; *p* < 0.001), and response urgency (β *=* 10.62; 95% CI, 9.85–11.40; *p* < 0.001) were all significant predictors of hand reaction time. The relationship between eye and hand responses was modulated by response urgency (β *=* 2.43; 95% CI, 1.67–3.18; *p* < 0.001) but not jump certainty. Figure 6C shows the trial-by-trial correlation between eye and hand reaction times for an exemplary participant (left column) and the correlation coefficients of all participants (right column) for each of the three urgency levels. Across conditions, eye and hand reaction times were positively but weakly correlated with correlation coefficients ranging from 0.1 to 0.6 (mean *r_low urgency_* = 0.27, mean *r_medium urgency_* = 0.36, and mean *r_high urgency_* = 0.30).

We found an effect of response urgency, *F*(2, 24) = 68.71, *p* < 0.001, η = 0.85, on hand vigor and a significant interaction between jump certainty and response urgency, *F*(2, 24) = 3.42, *p* = 0.049, η = 0.22. However, jump certainty did not systematically affect hand vigor, *F*(1, 12) < 2.2, *p* > 0.1, η < 0.2 (Figure 6D). Post hoc comparisons of the different urgency levels confirmed that participants responded more vigorously with increasing urgency. Specifically, hand vigor was greater in medium-urgency trials, *t*(25) = 8.7, *p_adjust_* < 0.001, *d* = 1.7, and high-urgency trials compared with low-urgency trials, *t*(25) = 11.3, *p_adjust_* < 0.001, *d* = 2.2. Also, hand vigor was greater in high-urgency compared with medium-urgency trials, *t*(25) = 9.4, *p_adjust_* < 0.001, *d* = 1.9.

Finally, we found no effect of jump certainty or response urgency on eye vigor (all *F* < 5.2, *p >* 0.5, η < 0.05) (Figure 6E). On a trial-by-trial level, we did not find a significant correlation between hand and eye vigor (*p* = 0.5). Thus, whereas hand movements became more vigorous as the urgency level increased, eye vigor was unaffected and not related to the hand response.

### Jump size differentially modulated eye and hand movement reaction times

We additionally investigated the effect of block type (constant jump size or mixed jump size) and jump size (3 cm or 6 cm) on eye and hand reaction times and vigor. For this analysis, we collapsed the data over certainty and urgency conditions. Hand response time was not affected by block type or jump size, *F*(1, 12) = 5.68, *p* = 0.03, η = 0.32, but there was a significant interaction, *F*(1, 12 = 8.42, *p* = 0.01, η = 0.41. Eye reaction time was affected by block type, *F*(1, 12) = 12.77, *p* = 0.004, η = 0.52, and jump size, *F*(1, 12) = 43.40, *p* < 0.001, η = 0.78, with no significant interaction. Specifically, participants initiated eye movements on average 5 ms earlier in blocks with constant jump size compared with mixed jump sizes and 13.5 ms earlier in 6-cm compared with 3-cm jump trials. These results indicate that, whereas the eyes responded earlier to larger target jumps, hand responses were relatively unaffected.

Hand vigor was not affected by block type, but we found an effect of jump size, *F*(1, 12) = 8.35, *p* < 0.001, η = 0.87, and a significant interaction, *F*(1, 12) = 23.08, *p* < 0.001, η = 0.87. Post hoc comparisons showed that participants responded more vigorously in the 6-cm jump trials compared with the 3-cm jump trials in the 3-cm and 6-cm blocks (*t* = 8.1, *p_adjust_* < 0.001, *d =* 2.2) and mixed jump size blocks (*t* = 8.9, *p_adjust_* < 0.001, *d =* 2.5). Eye vigor was not affected by block type but by jump size, *F*(1, 12) = 13.36, *p* < 0.003, η = 0.53, with participants responding more vigorously in the 6-cm compared with 3-cm jump trials. There was no significant interaction. These results indicate that the strength of the response (i.e., the vigor) was affected similarly for the eye and hand movement system.

## Discussion

**Figure 7.**
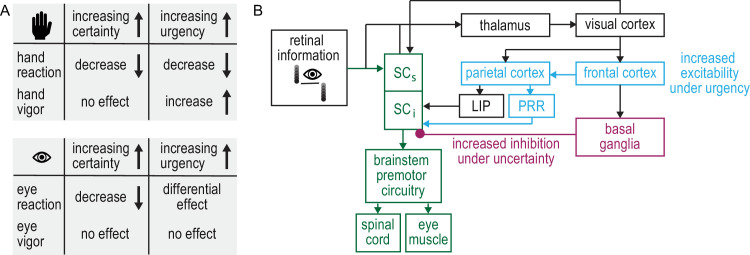
Summary of results and proposed neural modulations. (**A**) Effects of jump certainty and response urgency on hand (top) and eye (bottom) reaction time and vigor. (**B**) Simplified visuomotor circuit, highlighting areas involved in rapid responses. Retinal information is directly projected to the superficial layers of the superior colliculus (SC_s_) and transferred to the intermediate layers (SC_i_) that are involved in limb and oculomotor control (green). Retinal information also travels through the thalamus to the visual cortex and higher cortical areas. The parietal reach region (PRR) and the lateral intraparietal area (LIP) may drive signals to generate limb and eye responses in parallel.

In this study, we investigated the coordination of eye and hand responses during a rapid interception task. We found that eye and hand responses were differentially affected by various levels of jump certainty and response urgency (Figure 7A). Low-certainty conditions caused a delay in both eye and hand reaction times compared with high-certainty conditions. However, high-urgency conditions systematically led to earlier and more vigorous hand responses, whereas eye responses were largely unaffected by changes in response urgency.

### Eye–hand coordination depends on task demands

Goal-directed actions in natural environments require an integration of bottom–up sensory information and top–down cognitive goals (Fooken et al., 2023; Scott, 2016). Past research has shown that when reaching toward and manipulating stationary objects, eye and hand movements are highly coordinated in space and time (de Brouwer et al., 2021). When reaching toward stationary visual targets, eye and hand reaction times are generally moderately correlated (Fisk & Goodale, 1985; Prablanc, Echallier, Komilis, & Jeannerod, 1979). Interestingly, the correlation between eye and hand reaction times is weaker when the visual stimulus elicits rapid eye responses, such as in the gap paradigm, in which the initial fixation target is extinguished before the saccade target appears (Saslow, 1967). Moreover, the correlation between eye and hand responses is stronger when the task requires a cognitive component, such as memory-guided movements or movements to the opposite side of the cued target (Gribble, Everling, Ford, & Mattar, 2002; Sailer, Eggert, Ditterich, & Straube, 2000). The interception task used in this study elicited rapid eye and hand responses to visual perturbations. As in fast-paced or reactive stationary eye–hand coordination tasks, we found that rapid eye and hand responses when intercepting a perturbed moving target were only weakly correlated (*r* ≈ 0.3). Taken together, these results indicate that correlation between eye and hand movements depends on the visual information available and the cognitive task demands (Frens & Erkelens, 1991).

When intercepting or manually tracking moving objects, observers naturally keep their eyes on the target until they hit or catch it (Cesqui, Mezzetti, Lacquaniti, & d'Avella, 2015; Coudiere & Danion, 2024; Mrotek & Soechting, 2007). To compensate for high target speeds, the eyes typically track moving objects with a combination of smooth pursuit and saccadic eye movements (Binaee & Diaz, 2019; Fooken et al., 2016; Goettker, Brenner et al., 2019). In interception tasks that involve distinct visual predictions, such as a target bounce or spatially restricted interception zones, observers make anticipatory saccades to the future target location (Diaz et al., 2013; Fooken & Spering, 2020; Land & McLeod, 2000; Mann et al., 2019). Whereas better object tracking is correlated with higher interception accuracy when target motion is unpredictable and uncertain, eye–hand coordination is more flexible in situations of high motion predictability and visual certainty (Fooken et al., 2021). In our study, we found that participants did not reliably track the moving target prior to the target jump (Figure 4). Eye position between the time of target movement onset and the time of target jump varied extensively between participants, and the average eye velocity in the same time window was well below target velocity. These results indicate that participants did not attempt to keep their eyes on the moving target but instead kept their eyes relatively still to detect the target jump.

These results support the hypothesis that the oculomotor response was driven by the salient sensory event rather than by planning an anticipatory saccade to the future interception location (Figures 1B and 1C). Participants initiated saccades toward the location of the jumped target with latencies of ∼135 ms, which is slightly slower than human express saccades elicited in the gap paradigm (Fischer & Ramsperger, 1984) but faster than regular visually driven saccades (Irving, Steinbach, Lillakas, Babu, & Hutchings, 2006). Previous work has shown that saccade reaction times to moving targets depend on the visual features of the target, such as target velocity (Gellman & Carl, 1991; Ron, Vieville, & Droulez, 1989) or target contrast (Goettker, Braun, & Gegenfurtner, 2019). Importantly, these saccades are initiated with latencies of at least 170 ms and are able to compensate for target motion during saccade planning and execution (Engel, Anderson, & Soechting, 1999; Etchells, Benton, Ludwig, & Gilchrist, 2010; Gellman & Carl, 1991; Goettker, Braun et al., 2019; Ron et al., 1989). Here, we found that saccades in response to target jumps landed ahead of the moving target in low-urgency trials and behind the moving target in high-urgency trials. These results indicate that saccades were reactive, with limited time for anticipating the interception location (Findlay, 1983; Robinson, 2022). Interestingly, we found that participants did not make a second saccade to the interception location, even when participants had up to 500 ms to intercept following the target jump (low-urgency condition). This finding is in line with previous research showing that participants tend to suppress saccades shortly before intercepting moving objects (Goettker, Braun et al., 2019; Mrotek & Soechting, 2007). Overall, our results demonstrate that eye and hand responses are generally coordinated—but not necessarily correlated—to accomplish rapid goal-directed interceptions.

### Rapid visuomotor responses depend on target predictability

When reaching to or looking at visual targets, humans are able to quickly react to sudden changes in the target position (Goonetilleke et al., 2015; Gu, Wood, Gribble, & Corneil, 2016; Pruszynski et al., 2010; Wood, Gu, Corneil, Gribble, & Goodale, 2015). These fast orienting responses can be behaviorally measured across different movement systems, including the oculomotor and limb motor system. Previous work has shown that humans reliably elicit express visuomotor responses when intercepting a vertically moving target that is displaced behind an occluder (Kozak et al., 2020) and that these responses depend on temporal predictability (Contemori et al., 2021; Jakobs et al., 2009). Here, we used a similar experimental paradigm to investigate coordinated eye and hand responses to moving targets. We found that both eye and hand responses following the target jump were initiated earlier in trials in which a target perturbation was highly predictable (high-certainty blocks). Although the possible timings of the perturbation did not change across the experiment, participants had to inhibit a movement in 40% of the low-certainty blocks. These results suggest that the participants’ state of response readiness was reduced in the low-certainty condition, similar to an observed decrease in eye reaction time when catch trials are included in the gap paradigm (Kingstone & Klein, 1993; Paré & Munoz, 1996). Response readiness is also increased when targets rapidly move to a new location rather than being instantaneously displaced from one location to another (Reschechtko, Gnanaseelan, & Pruszynski, 2023), indicating that continuous motion prediction is important for rapid visuomotor responses.

In dynamic environments, humans are able to rapidly integrate contextual information to generate motor corrections (Kalidindi & Crévécoeur, 2023). The onset and strength of express visuomotor responses following visual perturbations depend not only on visual target features, such as stimulus luminance, orientation, or spatial frequency (Kozak, Kreyenmeier, Gu, Johnston, & Corneil, 2019; Marino et al., 2012; Veerman et al., 2008), but also on the behavioral context, such as target shape, texture, color, or contextual cues (Contemori, Loeb, Corneil, Wallis, & Carroll, 2022; Cross, Cluff, Takei, & Scott, 2019; Dimitriou, Wolpert, & Franklin, 2013; Veerman et al., 2008). Moreover, following mechanical perturbations, muscle responses and movement kinematics scale with the time available to respond (Crévécoeur et al., 2013; Poscente et al., 2021). Similarly, we found that hand responses were initiated earlier and more vigorously with increasing urgency, in their respective certainty conditions. The fact that we did not manipulate visual features at the time of perturbation suggests that hand response modulations across urgency levels were driven by the behavioral rather than visual context.

In contrast to the systematic effect of urgency on hand responses, we found that eye reaction time was lowest for medium-urgency trials compared with low- and high-urgency trials and that eye vigor was generally unaffected. Previous research has shown that saccade vigor is indicative of the subjective and economic value of visual targets in value-based decision-making tasks (Korbisch, Apuan, Shadmehr, & Ahmed, 2022; Reppert et al., 2015; Shadmehr, Reppert, Summerside, Yoon, & Ahmed, 2019; Yoon, Jaleel, Ahmed, & Shadmehr, 2020). Moreover, saccade vigor is increased when observers have to make a perceptual decision about a moving target in a manual interception task (Barany, Gómez-Granados, Schrayer, Cutts, & Singh, 2020). Perceptual decisions in urgent visuomotor tasks requiring saccade choices between two possible target locations have been shown to occur after an initial sensorimotor processing time of 90 to 180 ms (Salinas et al., 2019; Seideman, Stanford, & Salinas, 2018; Stanford & Salinas, 2021; Stanford, Shankar, Massoglia, Costello, & Salinas, 2010). Taken together, our results suggest that eye vigor is indicative of perceptual decisions and might be computed after an initial bottom–up sensory processing period.

### Neural mechanisms underlying rapid visuomotor responses

To successfully respond to a visual or mechanical perturbation within a few hundred milliseconds, the brain must rapidly transform visual input into motor output, a process that has been shown to involve subcortical circuits. In both the oculomotor and limb motor systems, the superior colliculus (SC)—a midbrain structure that interfaces sensory and premotor circuits—is involved in generating rapid orienting responses (Boehnke & Munoz, 2008; Cooper & McPeek, 2021; Corneil & Munoz, 2014; Gandhi & Katnani, 2011). In the oculomotor system, it has been shown that reduced activity of fixation-related neurons in the SC is linked to the generation of express saccades in the gap paradigm (Dorris, Klein, Everling, & Munoz, 2002; Dorris, Paré, & Munoz, 1997; Marino, Levy, & Munoz, 2015; Sparks, Rohrer, & Zhang, 2000). In the limb motor system, muscle responses to visual or mechanical perturbations can be detected as early as 90 ms (Corneil, Olivier, & Munoz, 2004; Gu et al., 2016; Pruszynski et al., 2010; Wood et al., 2015), and rapid muscle activity is highly correlated with neural activity in collicular reach cells (Philipp & Hoffmann, 2014; Stuphorn, Hoffmann, & Miller, 1999; Werner, Dannenberg, & Hoffmann, 1997). Moreover, when intercepting a moving target that abruptly shifts its position, muscle activity of human participants is modulated 90 to 110 ms after the perturbation, indicating that the speed of these rapid responses would likely involve subcortical pathways (Perfiliev, Isa, Johnels, Steg, & Wessberg, 2010).

Extensive work on visually guided eye movements has highlighted the subcortical mechanisms contributing to saccade generation and suppression (for a review, see Coe & Munoz, 2017). We propose that, in our task, two factors may modulate eye and hand responses: (1) a reduced inhibition, and (2) an increased excitability of the intermediate layers of the SC. Our observation that both eye and hand responses occurred earlier in high-certainty compared with low-certainty blocks suggests a common mechanism modulating visuomotor responses. We speculate that a change in certainty about an upcoming target jump affected inhibitory control from the basal ganglia to the SC (Figure 7B). Whereas inhibitory mechanisms were upregulated in low-certainty blocks and visuomotor responses had to be withheld in 40% of the trials, inhibition was reduced in high-certainty blocks. This potential regulation of inhibition affected both the oculomotor and limb motor systems similarly.

Our observation that eye and hand responses were differentially affected by urgency suggests that shared sensory information is used differently by the oculomotor and limb motor systems. We speculate that increasing urgency led to an increase in excitability along the limb motor pathway, possibly driven by top–down cortical processes (Contemori et al., 2023) (Figure 7B). Previous work on the neural control of eye–hand coordination has elucidated that both movement systems are controlled by shared early sensory and visuomotor processing areas (Battaglia-Mayer, Caminiti, Lacquaniti, & Zago, 2003; Crawford, Medendorp, & Marotta, 2004; Dean, Hagan, & Pesaran, 2012; Hwang, Hauschild, Wilke, & Andersen, 2014; Vesia & Crawford, 2012). More recently, the idea that the oculomotor and limb systems use the same task-specific sensory information, but operate in parallel to attain task goals, has been brought forward (Kang, Mooshagian, & Snyder, 2024). Our results support the idea of a parallel functional organization of eye–hand coordination stem that relies on shared sensory information but may serve separate behavioral goals (Figure 1).

### Eye–hand coordination depends on spatiotemporal task constraints

When controlling goal-directed actions, the human motor system must compensate for inherent delays that arise from transmitting and processing sensory input through neural pathways. For example, to accurately plan a saccade to a moving target, the oculomotor system has to rely on internal information about the trajectory and duration of the eye movement to compensate for the object motion (Schlag & Schlag-Rey, 2002). When planning a hand movement to a moving target, there is a similar delay of ∼100 ms to transform visual information into the limb motor command (Brenner & Smeets, 1997). However, unlike saccades, hand movements can be corrected online after the movement has been initiated and decisions about movement goals can be rapidly updated (Contemori et al., 2023; Nashed, Crévécoeur, & Scott, 2014). Previous work has proposed that initiating a goal-directed limb movement to visual targets is related to different stages of sensorimotor processing, including the detection of the target, movement selection, and execution (Smeets, Wijdenes, & Brenner, 2016). Correcting an ongoing movement to changes in target location relies on continuous updating of positional information. Such movement corrections occur at a relatively fixed latency of ∼100 ms and are made more vigorously when there is less time to respond to the perturbation (Oostwoud Wijdenes, Brenner, & Smeets, 2011). In our task, we cannot disentangle different stages of movement initiation and execution; however, we also found more vigorous limb responses when there was less time to respond (i.e., higher urgency). Moreover, we found that participants performed poorly in high-urgency trials, suggesting that the time to initiate the hand movement could not be further reduced. Overall, these results indicate that limb movement responses rapidly adapt to spatiotemporal task constraints.

We found that eye reaction times were shorter for targets that jumped 6 cm (9.2 deg) compared with 3-cm (4.6 deg). This is in contrast to previous findings in human and non-human primates showing that, when making visually guided saccades to targets that are further than 1 deg away, saccade latency increases with increasing target eccentricity (Hafed & Goffart, 2020; Kalesnykas & Hallett, 1994; Zhang & Fries, 2023). It should be noted that, in these studies, saccades were made to stationary targets at regular saccade latencies. Interestingly, short-latency saccades to predictable targets in the periphery are initiated earlier toward more eccentric compared with less eccentric targets (Cohen & Ross, 1977). Further, saccades to auditory targets are initiated earlier the more eccentric the target position (Gabriel, Munoz, & Boehnke, 2010). Taken together, these results suggest that salient sensory events may evoke even earlier orienting responses for targets of relatively high eccentricity.

## Conclusions

This paper highlights system-specific mechanisms guiding rapid eye and hand responses. We found that both eye and hand responses were similarly modulated by jump certainty, with reaction times decreasing when perturbations were highly predictable. However, we found that increasing levels of response urgency impacted eye and hand responses differently. Whereas hand responses scaled to the level of urgency, eye responses were relatively unaffected. We have proposed a framework that links our behavioral results to the neural mechanisms involved in the visuomotor coordination of the eye and hand. This framework can potentially offer a foundation for future research on motor actions in the face of unpredictable changes in our dynamic world.

### Citation diversity statement

Recent work in several fields of science has identified a bias in citation practices such that papers from women and other minority scholars are under-cited relative to the number of such papers in the field (Bertolero et al., 2020; Caplar, Tacchella, & Birrer, 2017; Chatterjee & Werner, 2021; Dion, Sumner, & Mitchell, 2018; Dworkin et al., 2020; Fulvio, Akinnola, & Postle, 2021; Maliniak, Powers, & Walter, 2013; Mitchell, Lange, & Brus, 2013; Wang et al., 2021). Here, we sought to proactively consider choosing references that reflect the diversity of the field in thought, form of contribution, gender, race, ethnicity, and other factors. First, we obtained the predicted gender of the first and last author of each reference by using databases that store the probability of a first name being carried by a woman (Dworkin et al., 2020; Zhou et al., 2020). By this measure (and excluding self-citations to the first and last authors of our current paper), our references are 7.14% woman (first)/woman (last), 8.16% man/woman, 19.39% woman/man, and 65.31% man/man. This method is limited in that (a) names, pronouns, and social media profiles used to construct the databases may not, in every case, be indicative of gender identity; and (b) it cannot account for intersex, nonbinary, or transgender people. Second, we obtained the predicted racial/ethnic category of the first and last author of each reference by referring to databases that store the probability of a first and last name being carried by an author of color (Ambekar, Ward, Mohammed, Male, & Skiena, 2009; Chintalapati, Laohaprapanon, & Sood, 2023). By this measure (and excluding self-citations), our references contain 4.67% author of color (first)/author of color (last), 23.22% white author/author of color, 17.89% author of color/white author, and 54.22% white author/white author. This method is limited in that (a) names and Florida voter data to make the predictions may not be indicative of racial/ethnic identity, and (b) it cannot account for Indigenous and mixed-race authors, or those who may face differential biases due to the ambiguous racialization or ethnicization of their names. We look forward to future work that could help us to better understand how to support equitable practices in science.
